# Strain and strain rate analysis of the right ventricle in patients with hypoplastic left heart syndrome

**DOI:** 10.1186/1532-429X-13-S1-P207

**Published:** 2011-02-02

**Authors:** Andrew W Hoyer, Thomas L Ratchford, Jiade Yu

**Affiliations:** 1 University of Virginia, Charlottesville, VA, USA

## Introduction

Assessment of right ventricular (RV) function in patients with hypoplastic heart syndrome (HLHS) is essential, but echocardiographic assessment remains largely subjective and RV volume quantification by CMR is time consuming.

## Purpose

To determine whether software designed for analysis of echocardiograms can be adapted to evaluate strain and strain rate of the RV in CMR images from patients with HLHS.

## Methods

Axial SSFP cine images from 10 patients status post Norwood-Sano procedure were analyzed using Siemens Vector Velocity Imaging software after each study's DICOM header had been modified. Longitudinal and radial strain and strain rate were calculated for the segment of RV myocardium at the right AV groove. 6 patients were female; median age was 3 months; mean weight was 5.5 kg.

## Results

**Figure 1:**
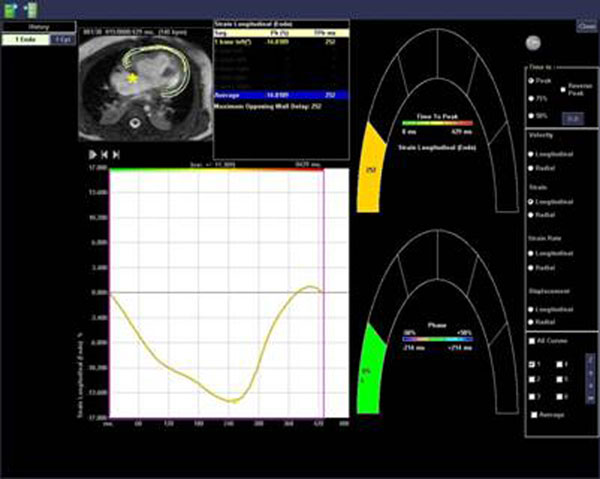
Screen shot from analysis program; curve of longitudinal strain is shown

The mean longitudinal strain of the RV at the AV groove is -7.1% while the mean strain rate is -0.9 s^-1^; the mean radial strain is 3.1% and the mean strain rate is -1.4 s^-1^. Figure 1

## Conclusions

Indices of myocardial performance (strain and strain rate) can be analyzed from SSFP cine images using a program designed to evaluate echocardiograms, offering the potential for improved assessment of the RV in patients with HLHS.

